# Varicose Veins

**Published:** 1882-11

**Authors:** 


### Varicose Veins.

The patient was an old man with beautiful examples in both legs. Since in the right leg there existed some phlebitis, it was not operated on, in the hopes that the inflammation might prove sufficient to obliterate the veins. On the right side the operation was done, without an anæsthetic, as follows: A piece of carbolized cat-gut, carried in a large curved needle, was passed through the skin about half an inch from the course of the vein and brought out at an equal distance on the other side. In this stitch the cat-gut passed under the vein. The needle is then re-introduced at the place of its exit and carried backwards between the vein and the skin, and made to emerge as near as possible to the place where it entered. The cat-gut is then firmly tied and the ends cut off, so that if possible the knot shall disappear under the skin. A dull needle is preferred by the operator, as the point is not so likely to emerge at an undesired place. The spray is used and a complete Lister dressing placed over the site of the operation. In this case the veins were tied in four places, one of which was above the knee, and the patient kept quiet in bed for a few days. It is claimed that this method has been very successful and the dangers are far less than by any other operation. It certainly has simplicity and ease of accomplishment to recommend it.—*The St. Louis Med. and Surg. Jour.*

